# Sulphur in the Treatment of Thread-Worms

**Published:** 1909-04-24

**Authors:** Thos. P. Flynn


### The General Practitioner's Column.

[Contributions to this Column are invited, and if accepted will be paid for.]

### SULPHUR IN THE TREATMENT OF THREAD-WORMS.

By THOS. P. FLYNN, M.B., L.R.C.P. & S. (Edinburgh and Glasgow).

As I have nowhere come across an allusion to sulphur as a treatment for thread worms, the following experiences may be worth mentioning.

The first case was that of a man between 40 and 50 years of age who suffered from some skin affection for which a weak sulphur ointment was applied locally, and about 9 grains of sulphur were given daily by the mouth. The skin condition improved, but what attracted the patient's attention most was that he no longer suffered from thread worms, which had caused him great annoyance for years. He did not mention this complaint in the first instance, because, having been treated on former occasions for it without avail, he had come to regard it as incurable. His statement at the time was a surprise to me; but as he described the worms so accurately, and the conditions to which they gave rise, I could not doubt but that the sulphur given internally had cured him.

Another case was that of a married woman, who took nine B.P. lozenges every day for ten or eleven days. She wrote saying that she had "solid" comfort while she was taking them. This woman lived at a great distance in the country, so that the remedy could not get a fair trial. A curious thing about this patient was that for two years while living in America she was absolutely free from the worms, but began to suffer again on her return home.

Nine grains of sulphur daily cured a man who suffered for a very long time from these parasites. His doctor prescribed injections of quassia for him, but, although he bought a syringe for the purpose, he never used it. Grown-up people are often shy of this remedy. A young child was completely rid of the worms in a week by 1½ grains of sulphur ter in die.

The above cases show that sulphur is at least worth a trial, especially as it is so easily administered. Injections are inconvenient, are disliked by every class of patient, and are not always successful. In country districts where there are no trained nurses, injections, to be efficient, must be given by the doctor himself, and this means time and trouble. Under such circumstances sulphur, as has been said, is worth a trial, but perhaps it would be more effective if given in larger doses than the above.

